# VDAC Genes Expression and Regulation in Mammals

**DOI:** 10.3389/fphys.2021.708695

**Published:** 2021-08-05

**Authors:** Federica Zinghirino, Xena Giada Pappalardo, Angela Messina, Giuseppe Nicosia, Vito De Pinto, Francesca Guarino

**Affiliations:** ^1^Department of Biomedical and Biotechnological Sciences, University of Catania, Catania, Italy; ^2^Section of Molecular Biology, Department of Biological, Geological and Environmental Sciences, University of Catania, Catania, Italy; ^3^we.MitoBiotech.srl, Catania, Italy; ^4^Section of Catania, National Institute of Biostructures and Biosystems, Catania, Italy

**Keywords:** VDAC mammalian genes, expression profile, gene structure, mitochondria, promoter methylation, core promoter elements, transcription factor binding sites

## Abstract

VDACs are pore-forming proteins, coating the mitochondrial outer membrane, and playing the role of main regulators for metabolites exchange between cytosol and mitochondria. In mammals, three isoforms have evolutionary originated, VDAC1, VDAC2, and VDAC3. Despite similarity in sequence and structure, evidence suggests different biological roles in normal and pathological conditions for each isoform. We compared *Homo sapiens* and *Mus musculus* VDAC genes and their regulatory elements. RNA-seq transcriptome analysis shows that VDAC isoforms are expressed in human and mouse tissues at different levels with a predominance of VDAC1 and VDAC2 over VDAC3, with the exception of reproductive system. Numerous transcript variants for each isoform suggest specific context-dependent regulatory mechanisms. Analysis of VDAC core promoters has highlighted that, both in a human and a mouse, VDAC genes show features of TATA-less ones. The level of CG methylation of the human VDAC genes revealed that VDAC1 promoter is less methylated than other two isoforms. We found that expression of VDAC genes is mainly regulated by transcription factors involved in controlling cell growth, proliferation and differentiation, apoptosis, and bioenergetic metabolism. A non-canonical initiation site termed “the TCT/TOP motif,” the target for translation regulation by the mTOR pathway, was identified in human VDAC2 and VDAC3 and in every murine VDACs promoter. In addition, specific TFBSs have been identified in each VDAC promoter, supporting the hypothesis that there is a partial functional divergence. These data corroborate our experimental results and reinforce the idea that gene regulation could be the key to understanding the evolutionary specialization of VDAC isoforms.

## Introduction

The presence of a family of β-barrel proteins in the mitochondrial outer membrane reflects that mitochondria originate from endosymbiotic bacteria. Indeed, in eukaryotic organisms, evolution has selected proteins that were able to confer ample permeability to the membrane while equipping them with structures that allow interaction with molecules bathing the membrane itself. With bacteria, where multiple porins are in place, it is also the case that there are more VDAC isoforms set up in eukaryotic mitochondria, likely to have derived from gene-duplication events. VDAC isoforms vary in their number in many species, one or two in yeast, three in mammals, and up to 10 in plants, proposing a hypothesis that each has differentiated biological roles (Al Bitar et al., 2003; Wandrey et al., 2004; Young et al., 2007; Homblé et al., 2012; Messina et al., 2012). The multigenic family of VDAC evolved, following the divergence of animal and plant kingdoms, which explains the unrelated number of isoforms in the two kingdoms. In mammals, duplication events gave rise to a gene family composed of three different isoforms. Among them, VDAC3 is considered the ancestor while VDAC1 is the most recently evolved (Saccone et al., 2003; Young et al., 2007; Wojtkowska et al., 2012). The recent divergence of VDAC paralog genes has conserved a similar structure in the gene and protein organization and function (Young et al., 2007; Messina et al., 2012). The discovery of VDAC genes, in a human and a mouse, established the starting point for studying VDAC by various *in vivo* and *in vitro* approaches. Different statements have been reached regarding the expression and distribution of the different isoforms, the structure of the proteins, the channels functionality, and their involvement in many cellular mechanisms (Raghavan et al., 2012; Shoshan-Barmatz et al., 2020).

The three-dimensional structure of VDAC was determined as a transmembrane channel, consisting of 19 β-strands with an additional N-terminal region, containing elements of α-helix (De Pinto et al., 2007, 2008; Bayrhuber et al., 2008; Hiller et al., 2008; Ujwal et al., 2008). Interestingly, a conserved folding was demonstrated in the mouse and the human VDAC1 proteins, enough to overlap almost perfectly the two proteins. Also VDAC2 was determined from a crystal structure (Schredelseker et al., 2014; Eddy et al., 2019), while VDAC3 was only modeled on VDAC1 structure, using predictive tools. Nevertheless, the structural organizations of VDAC2 and VDAC3 proteins are undistinguishable with VDAC1 (De Pinto et al., 2010).

Functional experiments of VDAC channels reconstitution in artificial membranes demonstrated that VDAC isoforms are able to form pores (De Pinto et al., 2008; Messina et al., 2012; Raghavan et al., 2012). While VDAC1 and VDAC2 have a similar conductance, ion selectivity and voltage dependence, VDAC3 channel activity revealed different features (Reina et al., 2010, 2020; Checchetto et al., 2014; Saletti et al., 2018; Queralt-Martín et al., 2020).

Functional conservation of VDAC mouse and human orthologs was also demonstrated in different cellular contexts: by complementation assay in yeast, lacking the endogenous porin (Δpor1) with either mouse or human recombinant VDAC1 (Sampson et al., 1997; Reina et al., 2010), in mice by VDAC gene interruption, and in human cells by VDAC gene interference. In VDACs knock-out mice, physiological defects in tissues, requiring high energy support, are linked to an altered structure and functionality of the mitochondria (Anflous et al., 2001; Sampson et al., 2001). Silencing VDAC isoforms in cellular models confirmed the compromised mitochondrial functionality in the regulation of essential mechanisms, such as ATP production (Okada et al., 2004), Ca^2+^ flux through the OMM (De Stefani et al., 2012), balancing ROS impairment (De Stefani et al., 2012), apoptosis, and autophagy (Koren et al., 2010).

Although the structure and the function of each VDAC isoform are, frequently, the subjects of the study, the promoter elements, the transcriptional factors, and the potential epigenetic control mechanisms affecting the transcriptional activity of VDAC genes have never been comprehensively analyzed. We have recently started to study human VDAC genes and their transcriptional regulation (Guarino et al., 2020; Zinghirino et al., 2020). In this work, we provide additional information about murine VDAC genes counterparts as they are available in the public databases. We combined these data with the scarcely available related literature arising from expression and function of proteins. At the end, a detailed comparative overview of human and mouse VDAC genes promoter structure, expression, and regulation is reported. *Homo sapiens* and *Mus musculus* species were chosen as representative of the most evolved mammals, presenting a high degree of genome synteny and organization. The information acquired in the mouse will be invaluable to extend our knowledge about human physiology and pathology.

## VDAC Gene Structure

The structural organization of mouse and human VDAC genes, of the coding sequence, and their chromosomal localization were defined by the late 1990s.

The VDAC1 protein sequence was determined by Edman degradation from human B cell hybridoma (Dermietzel et al., 1994). Later, VDAC1 and VDAC2 isoforms sequences were isolated, following a screening of human cDNA libraries (Blachly-Dyson et al., 1993). A few years later, two mouse VDAC coding genes were cloned from a brain cDNA library (Sampson et al., 1996a), and, almost simultaneously, a third mouse isoform, later defined as VDAC3, was identified from a heart cDNA library (Sampson et al., 1996b). The mouse VDAC genes were localized in chr11 5q (VDAC1) and in chr14 10q (VDAC2), respectively (Sampson et al., 1996a,b). Correspondence with the chromosomal localization of human genes was identified and correctly defined by FISH experiments that localized VDAC1 to the chr5 q31 and VDAC2 to chr10 q22 positions (Messina et al., 1999). To understand the correlation between the gene and the structural and functional features of VDAC protein isoforms (Ludwig et al., 1986; De Pinto et al., 1987), mouse and human VDAC genes were also characterized. VDAC genes were assembled, using the genome-walking approach, the terminus of each gene defined by 5′ and 3′ RACE-PCR, the exon/intron junctions identified, the polyadenylation signal localized, and the transcription initiation sites predicted, using TSSG and TSSW software available at that time (Sampson et al., 1997; Messina et al., 2000).

The organization of the three genes, their exon size and composition, and exon-intron junctions turned out to be very similar, suggesting recent gene duplication events of the isoforms. VDAC1 and VDAC3 have the same number of exons, nine, with comparable size. VDAC2 contains additional exon coding for the extension of 11 amino acids of the N-terminal sequence. Alignment of the nucleotide sequences revealed 70% of identity among the three isoforms of the same species and 90% identity between the isoforms of a human and a mouse, in particular in the coding region (Messina et al., 2012; Raghavan et al., 2012).

To summarize, three VDAC genes of comparable size, structures, and with a high degree of sequence conservation are present in the mouse and the human, confirming the syntenic relationship between the two species (Young et al., 2007; Raghavan et al., 2012).

## VDAC Alternative Splice Variants

**Figure 1:**
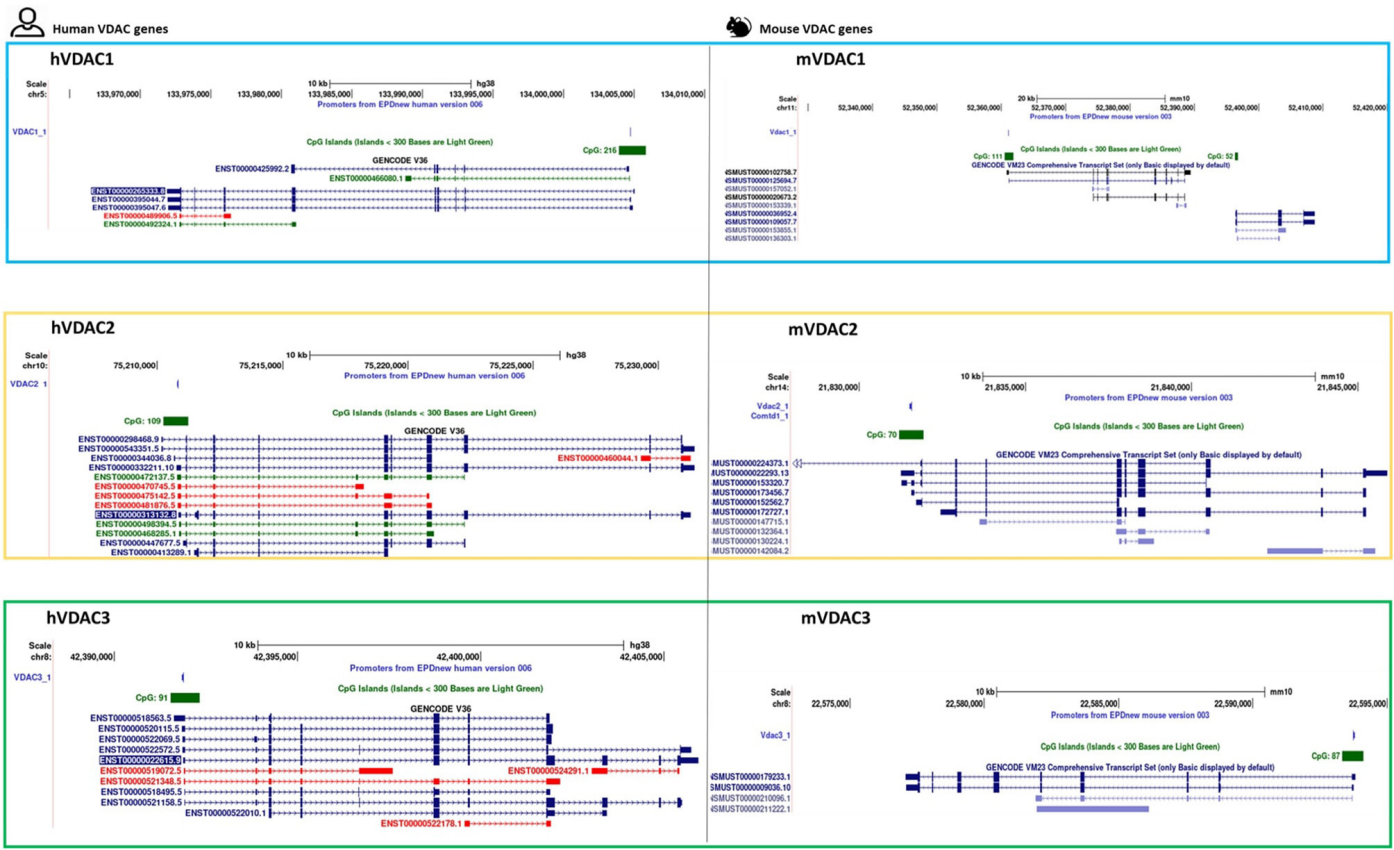
VDAC isoforms in human and mouse. A UCSC schematic overview of transcript variants, showing the location of CpG island and the promoter sequence from EPD v.006 (human) and v.003 (mouse). In the left panel, the colors conventionally used in the GENCODE V36 display of human transcripts show the protein coding sequence in blue, the noncoding mRNA in green, and the retained one in red. In the right panel, the mouse protein gene transcripts are shown in black and dark blue, while other RefSeq transcripts are in light blue according to GENCODE VM23 track configuration.

The results of VDAC genes sequencing and structure reconstitution in the human and the mouse led to the identification of alternative splicing variants. Although the identification of two additional exons in the 5′-untraslated region (5′-UTR) of the human VDAC1 gene suggested the generation of two transcript variants, the proteins encoded did not change the amino acid sequences derived from the canonical transcript (Raghavan et al., 2012; https://www.ensembl.org/index.html). The human VDAC2 gene holds an additional exon2 in the transcript, responsible for its longer N-terminal sequence of additional 11 amino acids (Ha et al., 1993). This feature of the second isoform was maintained during mammalian evolution, but it is absent, for example, in fish. As for the human VDAC3 gene, an alternative transcript was found in various tissues, starting from an extra ATG located between exon 3 and exon 4 and identified as a short, alternative exon, consisting of a single ATG codon. This alternative transcript is translated in a shorter, truncated protein, with features comparable to the two other isoforms. The expression of this transcript variant was also identified in a rat and a mouse (Sampson et al., 1998). We have recently analyzed the human transcript sequences available in the main public resources (ENSEMBLE and GenBank) through the Genome Browser retrieval system of UCSC (Kent et al., 2002; http://genome.ucsc.edu). In this work, we compared human information with those found about murine VDAC isoforms. Several transcript variants are annotated for each isoform, falling into three different groups: coding proteins; processed transcripts; or RNA involved in nonsense mediated decay (Zinghirino et al., 2020). With the exception of the VDAC3 gene, holding two transcript variants in the mouse and six in the human, a comparable number of different protein-coding mRNA can be accounted for the VDAC1 and VDAC2 isoforms in both species (Figure 1). The alternative mRNAs in human VDAC1 have the same exons composition but differ in 5′ and/or 3′ UTR lengths. Instead, in the mouse, the mRNA translated into the functional protein VDAC1 might be subjected to alternative splicing at the C-terminal end of the coding region to generate transcript variants for longer or truncated polypeptides. A similar observation might be made with human and mouse VDAC2 transcript variants as is suggested by their structural organization.

It is not known whether the identified alternative VDAC variants have any biological role. However, data collected by various collaborative projects available online report the expression of several mRNAs, including non-protein-coding transcripts. The variability of UTR sequences, as well as the presence of many non-coding RNAs, also supports the hypothesis that there are differentiated transcriptional regulatory mechanisms for each variant, depending on their cellular context.

## VDAC Gene Expression in Different Tissues

The very first information about the tissue-specific expression of VDAC isoforms in mammals was obtained by Northern blotting and RT-PCR experiments (Ha et al., 1993; Sampson et al., 1998). In general, a comparable level of VDAC transcripts in almost all human and mouse tissues was observed, with prevalence of VDAC1 isoform in every species. The highest amount of VDAC1 and VDAC2 transcripts was detected in brain, skeletal muscle, heart, digestive, and reproductive systems (Ha et al., 1993; Sampson et al., 1998). Interestingly, a peculiar expression of VDAC isoforms was observed in reproductive organs. Indeed, VDAC2 and VDAC3 are highly expressed in specific locations in sperm and oocyte, whereas VDAC1 was exclusively found in regions required to support gamete development, i.e., Sertoli cells (Hinsch et al., 2001; De Pinto et al., 2008). VDAC3 was also found to be essential for sperm motility; in the mice knockout of the VDAC3, the gene was associated with male infertility (Sampson et al., 2001). Furthermore, VDAC2 was detected in the acrosomal region where it finely regulates the balance between the life and death of these particular cells. In porcine oocytes, VDAC1 is localized in the plasma membrane and around the cortical area, whereas immunostaining of VDAC2 produced clusters of ring-like structures distributed over the cortical area in some stage oocytes (Cassará et al., 2010). Cryo-electron tomography images of mammalian sperms resolved the structure of mitochondrial wrap around the flagellum cytoskeleton and revealed a network of proteins, including VDAC2 and VDAC3. Their role is essential for maintaining the interaction between mitochondria and cytoskeleton and to stabilize sperms during motility and activation (Leung et al., 2021).

**Figure 2:**
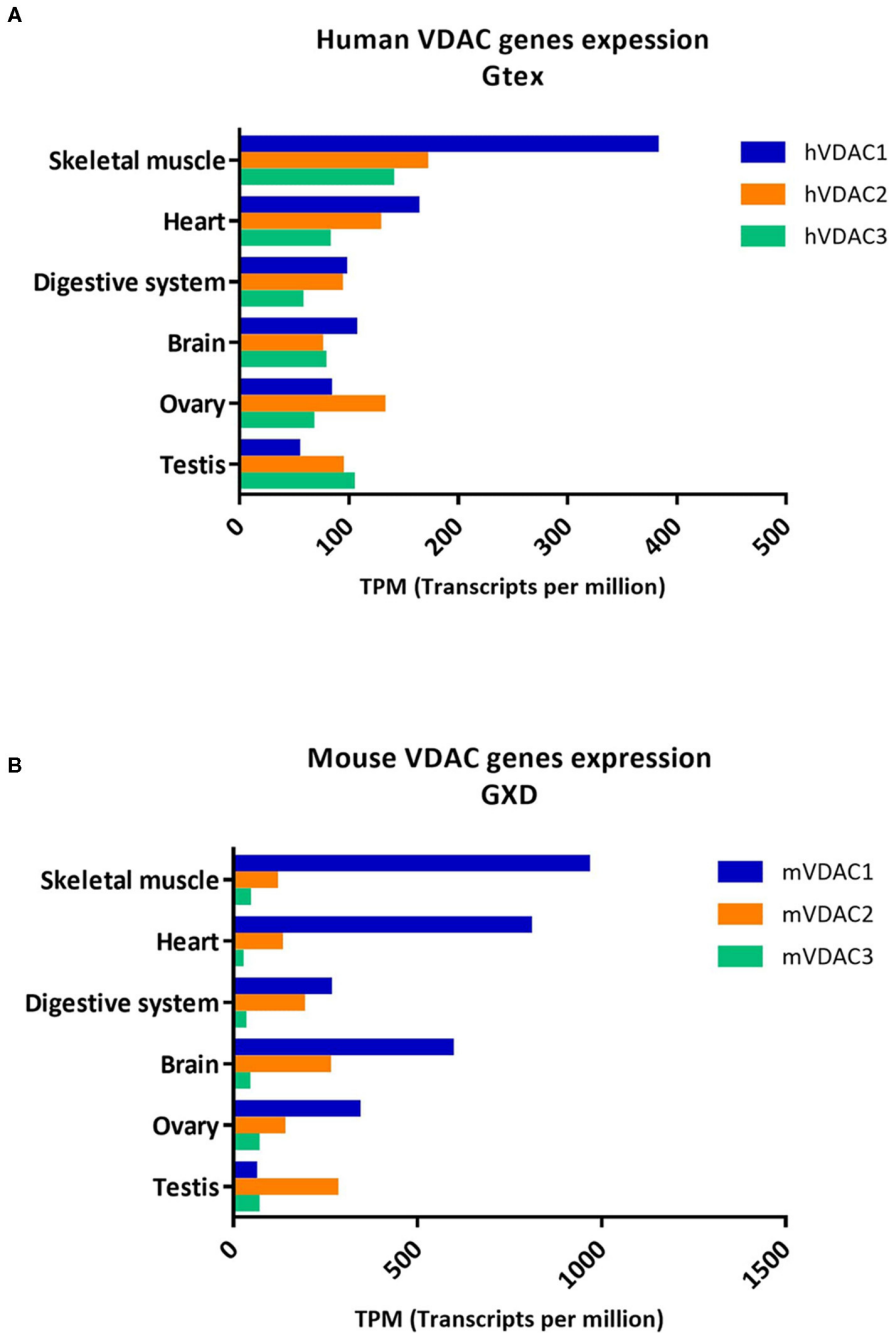
Human and mouse VDAC isoforms genes expression in different tissues. The expression level of human and mouse VDAC genes in the most common tissues as obtained from **(A)** Genotype-tissue expression (GTEx) related to expression Atlas of EMBL-EBI open science resource (https://www.ebi.ac.uk/gxa/home); and from **(B)** MGI-Mouse gene expression database (GXD) (http://www.informatics.jax.org/gxd).

To date, the information available on VDAC levels is primarily related to the investigation of changes between physiological and pathological conditions. These are corroborated by many results derived from large-scale genomic projects, high-throughput sequencing, and transcriptomics data. Recently, we have reported the expression profile of the human VDAC isoforms (Zinghirino et al., 2020) from the RNA-seqGTEx (Ardlie et al., 2015) and the RNA-seq CAGE RIKEN FANTOM 5 (FANTOM5) (Noguchi et al., 2017) Projects of Expression Atlas repository of EMBL-EBI (Papatheodorou et al., 2019). Here, we complement this information with the expression profile of VDAC mouse isoforms from the mouse gene expression database (GXD) (Baldarelli et al., 2021) and the FANTOM5 project repository (Figure 2 and Table 1). The aim is to evaluate the expression profiles of VDAC isoforms between the two species.

**Table 1:** Human and mouse VDAC expression in different tissues by RNA-Seq CAGE FANTOM 5 project (TPM values).

**Gene**	**Cerebellum**	**Diencephalon**	**Spinal cord**	**Heart**	**Lung**	**Pancreas**	**Colon**	**Ovary**	**Testis**
**VDAC genes expression by RNA-Seq CAGE FANTOM 5 (TPM values)**									
hVDAC1	NR	0.5	NR	0.5	NR	NR	0.6	NR	0.7
hVDAC2	1	2	1	1	0.8	0.8	1	0,8	1
hVDAC3	89	82	59	107	30	35	38	62	107
mVDAC1	42	83	70	NR	6	4	10	3	0.6
mVDAC2	NR	0,6	0,6	NR	NR	4	6	1	6
mVDAC3	35	39	45	NR	18	11	25	21	152

*RNA-seq cap analysis gene expression (CAGE) FANTOM5 data for the human and mouse VDAC gene were collected from the Expression Atlas repository (https://www.ebi.ac.uk/gxa/home). In the Table are the reported specific TPM values of each human VDAC isoform for representative tissues. NR: not reported in the dataset; TPM: transcripts per million*.

Analyzing VDAC expression profiles in these datasets, we can confirm that human VDACs are ubiquitously expressed in all tissues but mainly enriched in skeletal muscle and heart. The level of VDAC2 is comparable or slightly lower than VDAC1 mRNA, while VDAC3 is less expressed than the other two isoforms (Figure 2A). The results extracted from GTEx were also confirmed by real-time PCR experiments in HeLa cells with quantification of VDAC2 and VDAC3 mRNA levels relatively to the VDAC1 transcript (Messina et al., 2012; Zinghirino et al., 2020). The comparison of human VDAC transcripts obtained from RNA-seq data from the GTEx project with those collected from GXD revealed that mice also show prevalence of VDAC1 expression, the highest in skeletal muscle (Figure 2B). We also found a predominant level of VDAC1 transcripts over the other isoforms in the heart, digestive system, and ovary. However, in contrast to human RNA-seq data, where VDAC3 is the most expressed isoform in testis, in mice, we observed that VDAC2 is the most expressed in this tissue.

Surprisingly, the data set from the FANTOM5 project on humans overturned information from previous repositories, showing a very poor presence of VDAC1 transcripts, while VDAC3 and VDAC2 were much more abundant (Table 1). These data, indeed, would highlight for the first time the prevalence of VDAC3 gene transcription on other isoforms, reflecting its higher promoter activity. We do not know whether this is an experimental effect of the dataset accumulation process, since the RNA-seq methodology—based on cap analysis of gene expression adopted by the FANTOM5 consortium—aims to identify exclusively active TSS located at the 5′-end of transcribed mRNA, which are not necessarily associated with the entire protein coding transcripts (Zinghirino et al., 2020).

The results reported for mouse VDAC isoforms expression in different tissues by the FANTOM5 project are more homogeneous in terms of tissue enrichment and confirm a comparable order of magnitude between VDAC1 and VDAC3 isoforms expression, which are included in a range between 10 and 90 TPMI. VDAC2 is in a.5–6 TPMI range (Table 1).

One plausible explanation is that the level of each VDAC isoform expression is associated with the presence of the corresponding protein among tissues and, thus, to its putative specific biological role. For example, in mice, as well as in humans, VDAC3 is the most expressed isoform in testis and in tissues derived from different areas of the brain. In particular, in humans, VDAC3 expression is also reported to be high in other tissues as heart, cerebellum, diencephalon, spinal cord, and ovary. Among all the tissues tested in mice, VDAC2 is particularly highly expressed in the colon, testis, and pancreas, while, in humans, a prevalent level is registered in tissues or organs belonging to nervous system and circulatory apparatus. In these two last apparatuses and/or organs, VDAC1 expression is prevalent compared with the other tissues in both species.

## VDAC Gene Promoter Core Organization and Transcriptional Activity

The earliest information about the regulatory regions of mouse and human VDAC genes was reported when the gene structure of these protein families was described (Sampson et al., 1997; Messina et al., 2000).

All mouse and human VDAC genes lack TATA-box elements, showing, on the contrary, enrichment of GC content. The transcription initiation sites (TSS) were predicted in murine VDAC, and the 5′-UTR located downstream was cloned and studied for its transcriptional activity by the CAT gene reporter assay. Interestingly, the putative VDAC1 promoter was the less active among the three isoforms and lacked any significant activity in antisense orientation, while VDAC2 predicted promoter showed the highest transcriptional activation level (Sampson et al., 1997). Only recently, human VDAC gene promoters have been investigated to highlight their structural and functional features (Zinghirino et al., 2020).

**Figure 3:**
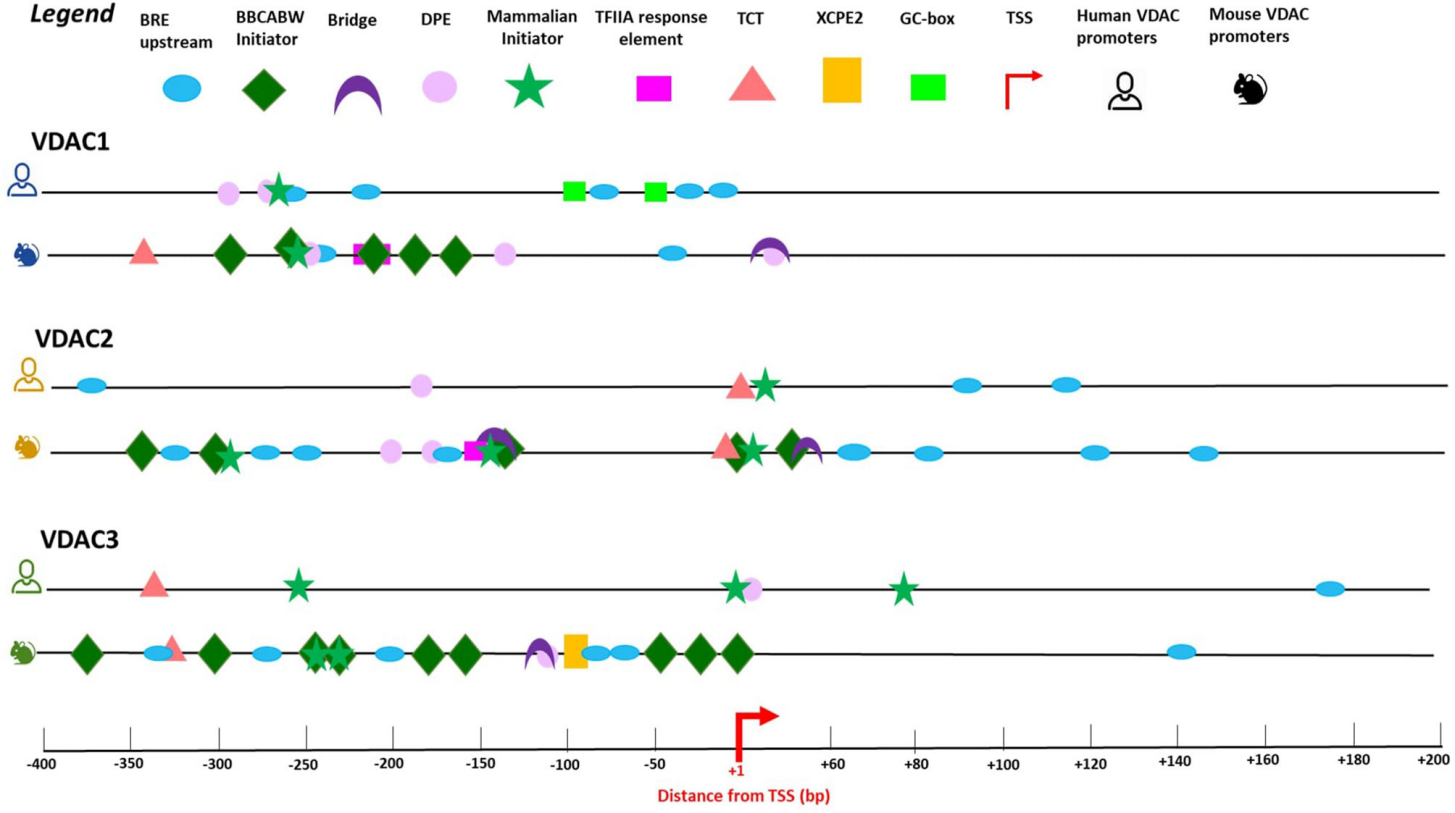
Promoter binding motifs and their location in human and mouse VDAC genes. The promoter sequences of mVDAC1, mVDAC2, and mVDAC3 genes used in this work cover a wider region around the TSS compared with the annotated core promoter in EPD database (EPD new v.006), ranging from 400bp upstream the TSS to 200bp downstream the TSS. The promoter elements identified by the predictive tool ElemeNT v.2 (http://lifefaculty.biu.ac.il/gershon-tamar/index.php/resources) illustrated in the legend box are B recognition element (BRE); BBCABW, Mammalian initiator, Bridge motif, downstream core promoter element (DPE), TFIIA response element, polypyrimidine initiator (TCT), and X core promoter element 2 (XCPE2). The TSS site is indicated by a red arrow. The scheme displays the similarity (conserved motifs) and differences in the structure and distribution of mouse promoter elements with the results of promoter elements in human VDAC genes (Zinghirino et al., 2020). Each promoter binding motif was chosen with highest scoring consensus based on functionally recommended scores of ≥0.1 for BBCABW Initiator, Human TCT and TFIIA response element; ≥0.01 for Bridge, DPE, MTE, XCPE 1, and XCPE 2. The human output might be less informative than mouse representation due to a quality operating filtering. Few elements were found with a maximum score due to a redundancy of human promoter elements.

For each VDAC isoform, we selected the main promoter region found in the eukaryotic promoter database (EPD) and confirmed its role through the analysis of the best predictors of transcription available in the UCSC genome browser: CpG island location, the RNA polymerase II binding site, the chromatin-state model, and the enrichment levels of the H3K4me1 and H3K4me3 histone marks (Figure 3).

We also investigated the promoter core structure and motif elements distribution involved in VDAC gene transcription. A general overview of VDAC promoter regions reveals that, coherently, with the analysis of mammalian promoter properties (Bajic et al., 2006), the functional promoter elements are more enriched in the upstream rather than downstream TSS region. Moreover, the different promoter elements identified in the two species suggest a specific set of transcription initiation active domains in the control of TSS.

VDAC genes core promoter organization is similar to that of most TATA-less human promoters of ubiquitously expressed genes, where the presence of abundant GC regions, alternative binding sites Inr, DPE, and BRE assure basal transcription. In murine VDAC genes, several BRE elements are localized in VDAC2 and VDAC3 mouse genes, and, interestingly, BBCABW initiator elements are abundant, but only in murine VDAC gene promoters (Figure 3). The Inr-like sequence BBCABW (where B = C/G/T and W = A/T) is the most abundant sequence in the vicinity of the TSS and detected in focused promoters in humans (Vongoc et al., 2017). A noncanonical initiation site termed “the TCT/TOP motif” (polypyrimidine initiator), the target for translation regulation by the mTOR pathway, oxidative, and metabolic stress (Nepal et al., 2020), was also identified in human VDAC2 and VDAC3 promoter and in all murine VDACs. This element is an intriguing fingerprint for the VDAC genes family, at least in mammals, since it is conserved in mice and humans, suggesting that transcriptional activation or the translational control exerted through these elements might be linked to a specific biological context. One recent discovery is a set of genes termed dual-initiation (DI) promoter genes; these hold non-canonical YC initiation that are proximal to or intertwined with the canonical YR initiation in the same core promoter region. This promoter architecture reflects two regulatory functions, which can generate distinct sets of RNAs with different posttranscriptional fates correlated with developmental stages or different responses to environmental stimuli (Nepal et al., 2020; Figure 3).

The promoter core analysis suggested that, with the exception of motifs predicted around the TSS of VDAC2 promoter, which is quite overlapping between the mouse and the human, the different promoter elements identified in the other isoforms suggest a specific set of transcription initiation active domains in the control of TSS.

We studied the transcriptional activity of the human VDAC promoter by gene reporter assays and, according to RNA-seq CAGEFANTOM5 results, we found that the VDAC3 promoter had the highest transcriptional activity, and VDAC1 promoter was, in contrast, the least active (Zinghirino et al., 2020). It could be hypothesized that these cells need to quantitatively regulate the level of VDAC3 mRNAs due to their different stability or to maintain a high level of transcripts in order to promptly respond to a particular stimulus.

## VDAC Gene Promoter Methylation Status

An interesting aspect of the gene promoter is the methylation status of the gene in question, crucial for a complete understanding of gene regulation. To date, the available information on the methylation status of VDAC genes is very poor. Therefore, we have extrapolated an overview of the genomic GC enrichment by UCSC Genome Browser. We further reported the methylation profile extracted by MethBank database (Li et al., 2018; https://bigd.big.ac.cn/methbank/), which provided data on VDAC genes methylation status in different tissues, cell types, and/or developmental stages (Table 2).

**Table 2:** Overview of CpG islands (CGIs) content in human and mouse promoters of VDAC genes.

**Gene**	**CpG Position**	**CpG size (bp)**	**% CpG**	**% C or G**	**ObsCpG/ExpCpG ratio**
**VDAC genes expression by RNA-Seq CAGE FANTOM 5 (TPM values)**					
hVDAC1	chr5:133339601-133341509	1,909	19.4	63.2	0.97
hVDAC2	chr10:76969993-76971002	1,010	21.6	71.6	0.84
hVDAC3	chr8:42249056-42249849	794	20.4	65.5	0.95
mVDAC1	chr11:52360505-52361866	1,362	16.3	62.6	0.83
mVDAC2	chr14:21831203-21831936	734	19.1	67	0.85
mVDAC3	chr8:22593328-22594117	790	22	68.2	0.95

*Comparative analysis of CGIs in VDAC promoter genes was performed by the gene regulatory track of UCSC Genome Browser, showing position, size, GC content (values expressed in percentage) and an observed/expected CpG ratio (Gardiner-Garden and Frommer, 1987). Releases of the human and mouse reference genomes were GRCh37/hg19 and GRCm38/mm10, respectively*.

In both species, the human and the mouse, VDAC1 gene owns the largest CpG island among isoforms, but both species are characterized by a comparable percentage of CpG as well as of C and G and of the observed/expected CpG ratio(Gardiner-Garden and Frommer, 1987). An extended CpG island in the promoter region is considered to be a feature of a more stable promoter activity compared with genes with a smaller CpG island, which needs to be specifically regulated (Elango and Yi, 2011). Although we suppose that VDAC1 is ubiquitously and stably expressed as a housekeeping gene, we also support the hypothesis that its transcriptional activity can be regulated in stress conditions when mitochondrial function needs to be assured in the cells (Guarino et al., 2020). For example, in tumor cells and in placental trophoblasts from patients with recurrent miscarriages, the increase of EPB41L4A-AS1 lnc-RNA induced the enhancement of VDAC1 promoter activity by histone modification. Indeed, the chromatin region where the VDAC1 gene is located showed increase in H3K4me3, mediated by the histone lysine methyltransferase SET1A and reduced interaction with histone deacetylase HDAC2. In this situation, EPB41L4A-AS1 lnc-RNA is an important regulator of reprogramming tumor and trophoblast cells metabolism, since it can activate oxidative metabolism by enhanced mitochondrial function (Liao et al., 2019; Zhu et al., 2019).

Furthermore, we extracted the methylation profile of VDAC genes from MethBank Database for a panel of tissues and cell lines in humans and for a panel of cell types at different development stages in mice (Table 3). The level of CG methylation of the human VDAC genes promoter revealed that the VDAC1 promoter is less methylated than the other two isoforms. Interestingly, VDAC2 promoter shows the highest methylation level, especially in the brain, muscle, and heart. The low degree of VDAC1 promoter methylation in all the tissues considered may suggest the predominant expression of this isoform, allowing it to accomplish its main role of exchanging mitochondrial metabolites. The average methylation levels of mouse VDAC genes across different samples of a specific developmental stage were also investigated. As a general rule, DNA methylation pattern changes dynamically during development, showing a very low level in primordial germ line and after fertilization while a re-methylation process is found in the later stage of germ cell and embryo development. We found that comparable methylation percentage is associated with VDAC2 promoter, gene body, and the downstream gene region, while, in VDAC1 and VDAC3, the promoter methylation status can be absent (Zeng and Chen, 2019). The higher methylation status of mouse VDAC2 gene is also confirmed in two particular cell types, oocyte and sperm, where VDAC proteins play a specialized role, revealing that this gene might be subjected to fine regulation affected also by epigenetic mechanisms. In support of this hypothesis, in human males, abnormal methylation of CpG island of VDAC2 promoter determined a decrease of VDAC2 expression, leading to lack of sperm motility and male infertility (Xu et al., 2016).

**Table 3:** Average methylation levels of VDAC genes across different normal human and murine samples from single-based resolution methylomes (SRMs) provided by MethBank (v.4.1).

**Gene**	**Brain**	**Muscle**	**Heart**	**Digestive system**	**Placenta**			
**Average methylation levels of VDAC promoters (β-value of 0–1)**								
hVDAC1	0.26	0.23	0.24	0.22	0.31			
hVDAC2	0.63	0.62	0.61	0.57	0.52			
hVDAC3	0.42	0.42	0.40	0.35	0.32			
**Gene**	**2-cell**	**4-cell**	**E13**	**E6.5**	**E7.5**	**ICM**	**Oocyte**	**Sperm**
mVDAC1	0.11	0.08	0.01	0.08	0.12	0.03	0.07	0.01
mVDAC2	0.48	0.41	0.02	0.19	0.25	0.17	0.69	0.2
mVDAC3	0.05	0.02	0.01	0.09	0.11	0.01	0.01	0.07

*Methbank (http://bigd.big.ac.cn/methbank) contains the SRM profiles from a wide range of cell/tissue lines in human and from mouse embryonic development stages. According to MethBank, the promoter region is defined as the region 2000 bp upstream of transcription start site (TSS) for animals. Furthermore, the average methylation data are calculated as β-Value that reflects the methylation intensity at each CpG site. β-Values of 0–1 (represented from 0 to 1) indicate signifying percent methylation, from 0 to 100%, respectively, for each CpG site (Dhas et al., 2015). We specifically selected human and mouse SRMs of the methylation level in the VDAC gene promoter. On the left, the human SRM profiles of tissues associated with the specific expression pattern of VDAC genes as discussed in Zinghirino et al. (2020) are shown*.

## VDAC Gene Promoter and Transcription Factor Binding Sites Distribution

Having isolated mouse and human VDAC genes, we investigated their basic elements. The starting points of the transcription were set up and defined the predicted promoters as the upstream region, containing the regulatory elements of the expression of the genes.

Using the available bioinformatic approaches, the binding of some transcription factor was predicted and the conservation of their sequence into the promoter of VDAC genes of human and mouse observed (Sampson et al., 1997; Messina et al., 2000). A database search for transcription factors binding motifs revealed the presence of several SP1 and AP-2 sites, but the most significant identified TFBS were a sterol repressor element (SRE), an SRY, the testis-determining factor, and nuclear respiratory factor 2 (NRF-2) binding sites, which respectively led to hypothesize the involvement of VDAC1 in cholesterol traffic, sex determination, and mitochondrial biogenesis (Sampson et al., 1997; Messina et al., 2000). With the exception of these few notes, the regulative regions of VDAC genes and the mechanisms associated with their expression were not thoroughly investigated yet.

However, understanding the mechanisms triggering VDAC genes transcription may highlight the biological role of each isoform inside the cells and in different biological contexts. Thus, in our recent analysis of human VDAC promoter genes, we laid the foundation for studying the regulatory mechanisms of VDAC isoforms expression. In order to reveal the TFs that bind to the promoter of genes, JASPAR and UniBind were used for TFBS enrichment. We defined the distribution of TFBSs by scanning VDAC promoter sequences with three different genomic suites (Genomatix, JASPAR, UniBind) and crossed the results with the data experimentally validated by ChIP-Seq (ENCODE Project v3). With this approach, we found the most relevant families of TFs regulating VDAC genes expression (Zinghirino et al., 2020), confirming the central role of VDACs in regulating mitochondrial function in fundamental cell processes. Indeed, in all three VDAC promoters, the majority of identified TFs classes participate in many similar activities but are prevalently involved in cell life and death, differentiation and development, and metabolism regulation (Grandori et al., 2000; Thiel and Cibelli, 2002; Liu et al., 2004; Niederreither and Dollé, 2008; Qu et al., 2010; Woo et al., 2011; Kim et al., 2017).

Using the same approach, we integrated this analysis with the characterization of TFBS located on mouse VDACs promoters. The comparison between the human and the mouse revealed both functional similarities and divergence of regulatory regions.

**Figure 4:**
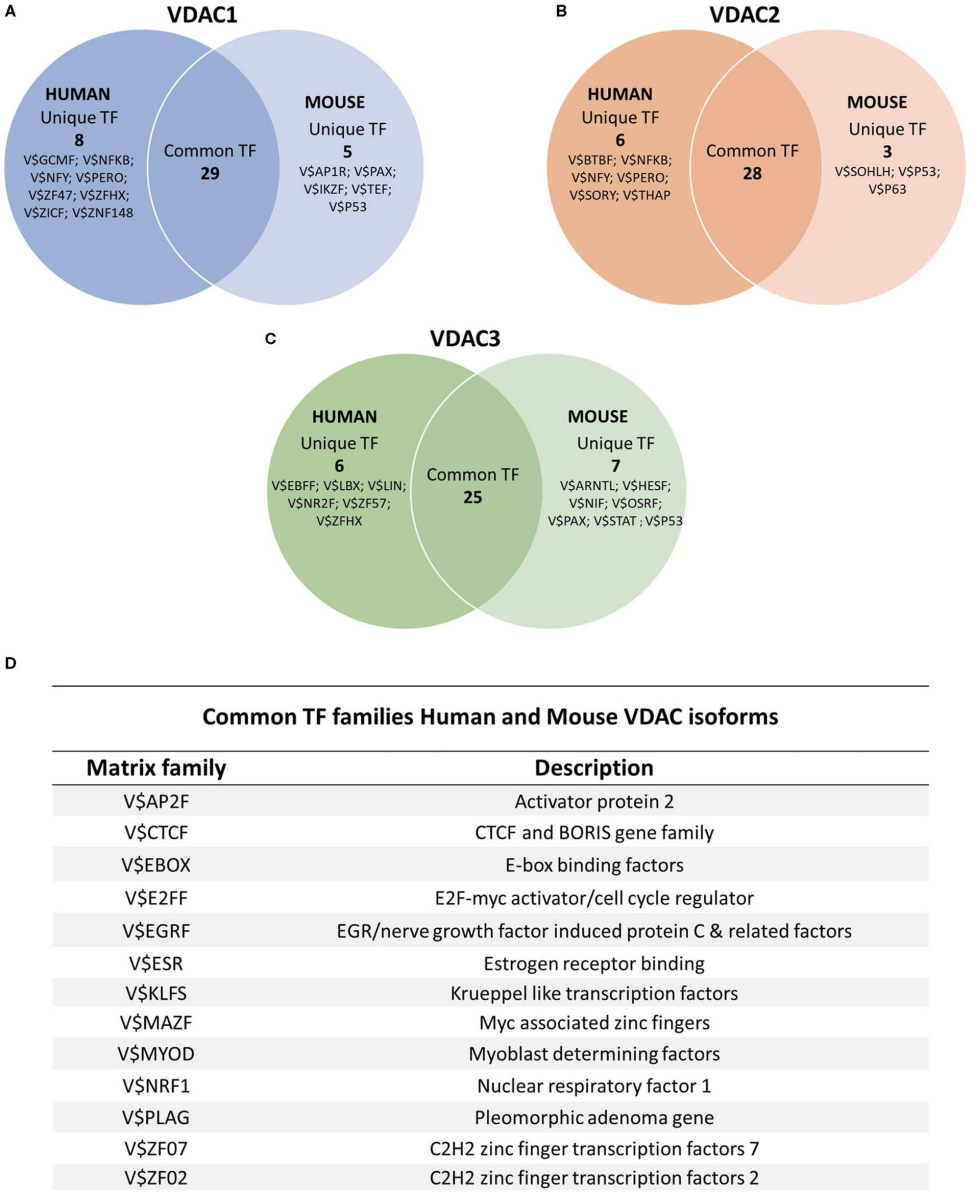
TFBS distribution on human and mouse VDAC promoters. The sequences of human and the promoter of mouse VDAC genes were analyzed by using JASPAR (http://jaspar.genereg.net/) and UniBind (https://unibind.uio.no/) databases, and the results were overlapped to find the most relevant TF families. A comparative analysis of common and unique TFBSs for each individual VDAC isoform of human and mouse was performed. Venn diagrams showed the number of common and unique TFBS clusters between human and mouse genes in **(A)** VDAC1, **(B)** VDAC2, and **(C)** VDAC3 promoters. **(D)** The results of the common TFBS families obtained from the comparative analysis of VDAC isoforms for each species were overlain to obtain the most evolutionarily conserved transcription factor families between a human and a mouse. In the Table, the Matrix family and the description of the main TFBS families shared among the three isoforms in both species are reported.

Comparing the VDACs genes of the two species, we brought to light a common fingerprint for main transcription factors; it comprises E2FF (E2F-myc cell cycle regulator), EBOX (E-box binding factors), KLFS (Krueppel-like transcription factors), NRF1 (nuclear respiratory factor 1), EGRF (EGR/nerve growth factor-induced protein C and related factors), MYOD (Myoblast determining factors) families, controlling cell survival, apoptosis, proliferation, differentiation, development, and metabolism regulation. The overlap of conserved transcription motifs reveals that there was a common evolutionary path of VDAC isoform regulatory regions in mammals. Meanwhile, other distinctive TF families exclusively characterize mouse or human VDAC promoters; however, in line with the previous observation, all of them fall back on those regulators involved in the main biological processes of growth, differentiation, and development in which cell, tissue, and organs need to be controlled in physiological and pathological conditions (Figure 4; Supplementary Tables 1–6).

Interestingly, we found that all mouse VDAC promoters bear specific motifs in their sequences recognized by the family of the p53 tumor suppressor transcription factors, including p63 in addition to p53. Their role in cell death, cell-cycle arrest, senescence, and metabolic regulation in response to cellular stress is well-known to affect cancer development (Chillemi et al., 2017; Fisher et al., 2020). Conservation of these TF families in mouse VDAC promoters allows us to hypothesize that the VDAC proteins are crucial in mitochondrial metabolism and in the balance of cell life and death. These functions were probably selected during the evolution of the VDAC gene family in mammals, and a more specialized role was acquired by each VDAC isoform in humans.

## VDAC Isoforms Specific Transcription Factors

There are several examples of experimental evidence reported in the literature that confirm the crucial role that VDAC isoforms play in specific tissues or, in particular, biological contexts, but their transcriptional specificity and programming have not been fully elucidated until now.

The identification of specific transcriptional regulators in the human VDAC1 promoter has demonstrated a prevalent role for this isoform in mitochondrial function in conditions that force cells to keep the energy balance of mitochondria (Fang and Maldonado, 2018; Shoshan-Barmatz et al., 2020). In particular, we found families of regulators required for cell response to hypoxia (V$AHRR: AHR-arnt heterodimers and AHR-related factors; Labrecque et al., 2013), mitochondrial biogenesis and redox homeostasis (V$ETSF: human and murine ETS1 factors; V$PBXC: Pre-B cell leukemia transcription factor; Hayes and Dinkova-Kostova, 2014; Morgan and Pandha, 2020), and cell stress response (V$HEAT: heat shock factors; Morimoto, 2011). Similarly, after searching transcription factor binding site (TFBS) in mouse VDAC1 promoter, we confirmed the hypothesized role of VDAC1 and highlighted the presence of specific motifs recognized by transcription factors regulating growth (V$AP1R, activator protein 1; Hess et al., 2004) and developmental processes (Watkins et al., 2009) (V$TEAD: TEA domain family member 1; Zhou et al., 2016), which are mainly involved in tumorigenesis. Inspired by our bioinformatic prediction of TFBSs distribution, we wanted to investigate the regulation of human VDAC1 gene expression in metabolic stress conditions (Guarino et al., 2020). The discovery of several binding sites for the NRF-1 and HIF-1α, transcriptional regulators involved in metabolic stress caused by nutrients (Scarpulla, 2006, 2012), and oxygen deficiency (Majmundar et al., 2010; Iommarini et al., 2017) in the VDAC1 promoter suggested a pivotal role for this isoform in the regulation of mitochondrial metabolism and energetic balance in cell adaptive response. We experimentally demonstrated that NRF-1 (nuclear respiratory factor 1) and HIF-1α (hypoxia-inducible factor 1-alpha) act as transcriptional activators of the VDAC1 promoter and can support the modulation of regulation of the VDAC1 promoter, following serum starvation and hypoxia (Guarino et al., 2020). Our results also suggested that the VDAC1 promoter activation in stress conditions is probably controlled by a more complex transcriptional apparatus, involving the cooperation of other transcriptional regulators. With the bioinformatics predictive tools and the ChIP-Seq Peaks data in ENCODE project validation, we predicted several transcription factors binding sites involved in mitochondrial biogenesis (CREB: cAMP response element-binding protein; SP1: specificity protein 1; ETS: human and murine ETS1 factors; CREM: cAMP response element modulator; ATF7: activating transcription factor 7; NFE2L2: nuclear factor erythroid 2-related factor 2; E2F), which might cooperate with NRF-1 or HIF-1α for the regulation of VDAC1 transcription. According to these data, it has been recently reported that the CREB effector of MAPK/ERK signaling pathway triggers the increase of VDAC1 transcription to activate apoptosis induced by H2O2 in cardiac microvascular endothelial cells. Such overexpression can be reestablished at a basal level when melatonin is used as an antioxidant to repair the injury (Xing et al., 2019). The experimental observation of VDAC1 transcriptional regulation in stress conditions confirms the importance of this protein in maintaining the correct balance in cell life and death by assuring the functionality of the mitochondria (Fang and Maldonado, 2018; Shoshan-Barmatz et al., 2020).

Analysis of the human VDAC2 promoter highlighted the presence of factors specifically involved in the development of specialized tissues and the organogenesis process mainly related to nervous system genesis (V$NEUR: NeuroD, Beta2, HLH domain; Kageyama et al., 2019) and growth [V$CLOX: CLOX and CLOX homology (CDP) factors; Li et al., 2010]. Corresponding findings arose from the analysis of the mouse VDAC2 promoter; however, different TFs are present, like V$SOHLH (spermatogenesis and oogenesis basic helix-loop-helix; Suzuki et al., 2012) and V$ZFXY (Zfx and Zfy-transcription factors; Fang et al., 2014), both associated with sex tissue development. The role of VDAC2 in this particular aspect is supported by the literature. GATA1 (GATA-binding factor 1) and MYBL2 (cellular and viral myb-like transcriptional regulators) were identified as regulators of the VDAC2 promoter in developing porcine ovary, determining its upregulation to inhibit autophagy through the interaction with BECN1 (Beclin 1) and BCL2L1 (Bcl-2-like 1). The region of the VDAC2 promoter involved in the regulation of ovary development identified in the *Sus scrofa* promoter is highly conserved in mice. This observation highlights the protective role of VDAC2 in ovary development in all mammals. An interesting finding is the identification of two different haplotypes associated with this promoter region, which determine a difference also in the transcriptional activity of the VDAC2 promoter (Yuan et al., 2015).

With regard to the human VDAC3 isoform, the identified TFBSs belong to various families, but those involved in organogenesis [V$FOX: Forkhead (FKH)/Forkhead box (Fox); V$LBXF: Ladybird homeobox (lbx)] (Friedman and Kaestner, 2006; Jennings et al., 2019) in the development of germinal tissues and sex determination (V$SOHLH; Suzuki et al., 2012) are the most abundant. The bioinformatic distribution of TFBS in the mouse VDAC3 promoter shows enrichment for some specific motifs required for mitochondrial stress response (V$HESF: Vertebrate homologs of enhancer of split complex; Ardlie et al., 2015), organs development (V$OSRF; Otani et al., 2014), tumor suppressor (V$P53; Sullivan et al., 2018).

The promoter analysis of motifs elements involved in gene transcriptional activity and the identification of the regulators by the TFBS distribution, mainly delineate, in the VDAC gene, clusters of evolutionarily conserved transcription motifs. However, we also simultaneously found some distinctive traits corresponding to TF families regulating mouse or human specific VDAC genes. From the biological point of view, the overall picture emerging from the analysis of the specific TFBS, characterizing VDAC promoters, reports a less undifferentiated role of VDAC isoforms in mice. All the isoforms in mouse seem to be involved in the mechanism of cell stress response, in particular in tumorigenesis, while in humans, this role has been assigned to VDAC1.

## Conclusions

The activity of genes is a hallmark of the role of the proteins they encode. This aspect is even more relevant when analyzing a family of protein isoforms. In this work, we attempted to look for a nexus that could explain the evolution of three isoforms of the pore-forming protein VDAC and the need in the higher-evolved organisms to maintain such diversity. The comparison of promoter features of VDAC1-3 genes in close but different species as *H. sapiens* and *M. musculus* revealed that same functions, at least in terms of abundance/reduction of gene expression, are prevalently conserved among these two organisms, even though with some differences. The difference we found among human VDAC isoforms (Zinghirino et al., 2020) were mainly confirmed in mice, thus strengthening our feeling that the VDAC isoforms play different roles in the cell. The wealth of information stored in public databases appears a complex jungle. Our goal is to locate the classic needle in the haystack. With this work, we began to achieve this goal.

## Author Contributions

FG conceived the work. FZ, XP, AM, GN, VDP, and FG wrote the manuscript. FZ and XP equally performed the data collection and analysis. All authors contributed to the article and approved the submitted version.

## Conflict of Interest

AM, VDP, and FG have an intellectual collaboration with the company, we.MitoBiotech.srl. The remaining authors declare that the research was conducted in the absence of any commercial or financial relationships that could be construed as a potential conflict of interest.

## Publisher's Note

All claims expressed in this article are solely those of the authors and do not necessarily represent those of their affiliated organizations, or those of the publisher, the editors and the reviewers. Any product that may be evaluated in this article, or claim that may be made by its manufacturer, is not guaranteed or endorsed by the publisher.

## Abbreviations

AHRR: AHR-arnt heterodimers and AHR-related factors
AP1/AP1R: activator protein 1
AP-2: Activator protein-2
ATF7: activating transcription factor 7
BBCABW initiator: B = C/G/T, W = A/T
BCL2L1: Bcl-2-like 1
BCL6: B-cell lymphoma 6 protein
BECN1: Beclin 1
BEDF: BED subclass of zinc-finger proteins
BRAC: Brachyury gene, mesoderm developmental factor
BRE: B recognition element
CDXF: vertebrate caudal related homeodomain protein
CLOX: CLOX and CLOX homology (CDP) factors
CpG: 5′—C—phosphate—G-−3′
CREB: cAMP response element-binding protein
CREM: cAMP response element modulator
DB: database
DI: dual initiation
DPE: downstream promoter element
E2FF: E2F-myc activator/cell cycle
EBOX: E-box binding factors
EGRF: epidermal growth factor receptor
EPD: eukaryotic promoter database
ER: endoplasmic reticulum
ETSF: human and murine ETS1 factors
FANTOM: functional ANnotation of the mammalian genome
FOX: Forkhead (FKH)/Forkhead box (Fox)
GATA1: GATA-binding factor 1
GTEX: genotype-tissue expression
GXD: gene expression database
HEAT: heat shock factors
HESF: vertebrate homologs of enhancer of split complex
HMG: high-mobility group family
IKZF: Ikaros zinc finger
Inr: initiator
KLFS: Krueppel-like transcription factors
LBXF: ladybird homeobox (lbx)
lnc-RNA: long noncoding RNA
MOM: mitochondrial outer membrane
MYBL2: cellular and viral myb-like transcriptional regulators
MYOD: myogenic differentiation 1
NEUR: NeuroD, Beta2, HLH domain
NFE2L2: nuclear factor erythroid 2-related factor 2
NRF-1: nuclear respiratory factor 1
NRF-2: nuclear respiratory factor 2
Obs/Exp CpG: observed/expected CpG
OSRF: odd-skipped related factors
PBXC: Pre-B cell leukemia transcription factor
RACE: rapid amplification of cDNA ends
SOHLH: spermatogenesis and oogenesis basic helix–loop–helix
SP1: specificity protein 1
SRE: sterol repressor element
SRY: sex-determining region Y
TCT/TOP motif: polypyrimidine initiator
TEAD: TEA domain family member 1
TF: transcription factor
TFBS: tanscription factor binding site
TSS: transcription start site
VDAC: voltage-dependent anion channel
XCPE2: X core promoter element 2
ZFXY: Zfx and Zfy-transcription factors.
