# Supplementary material for: VDAC Genes Expression and Regulation in Mammals
Source: Front Physiol. 2021 Aug 5;12:708695. doi: 10.3389/fphys.2021.708695 (PMC8374620; doi:10.3389/fphys.2021.708695)

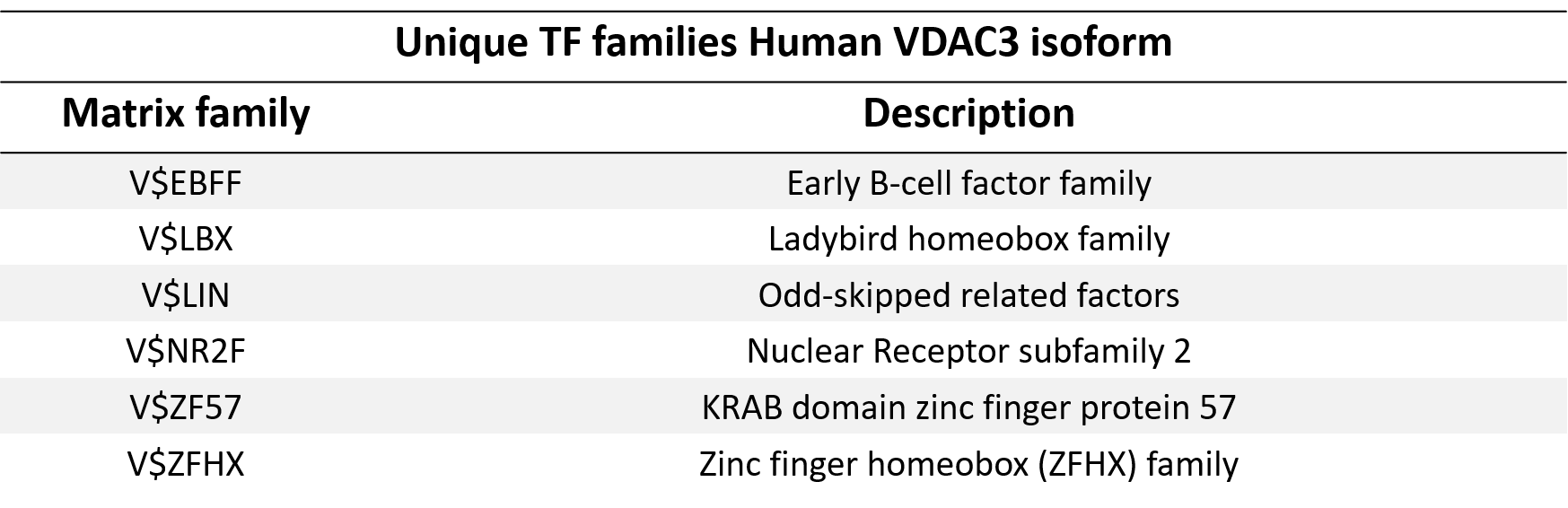


**Table S1. Matrix family and the description of the unique TFBS families in human VDAC1 promoter.**


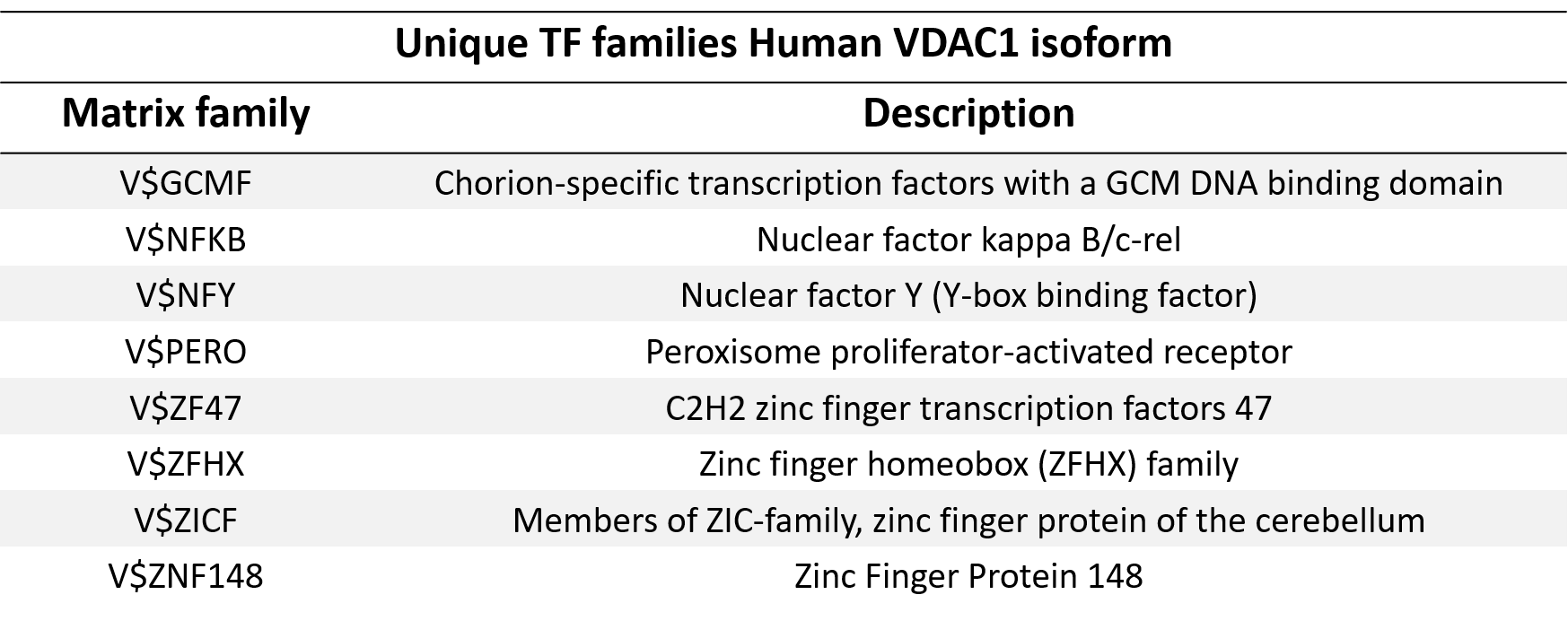


**Table S2. Matrix family and the description of the unique TFBS families in human VDAC2 promoter.**


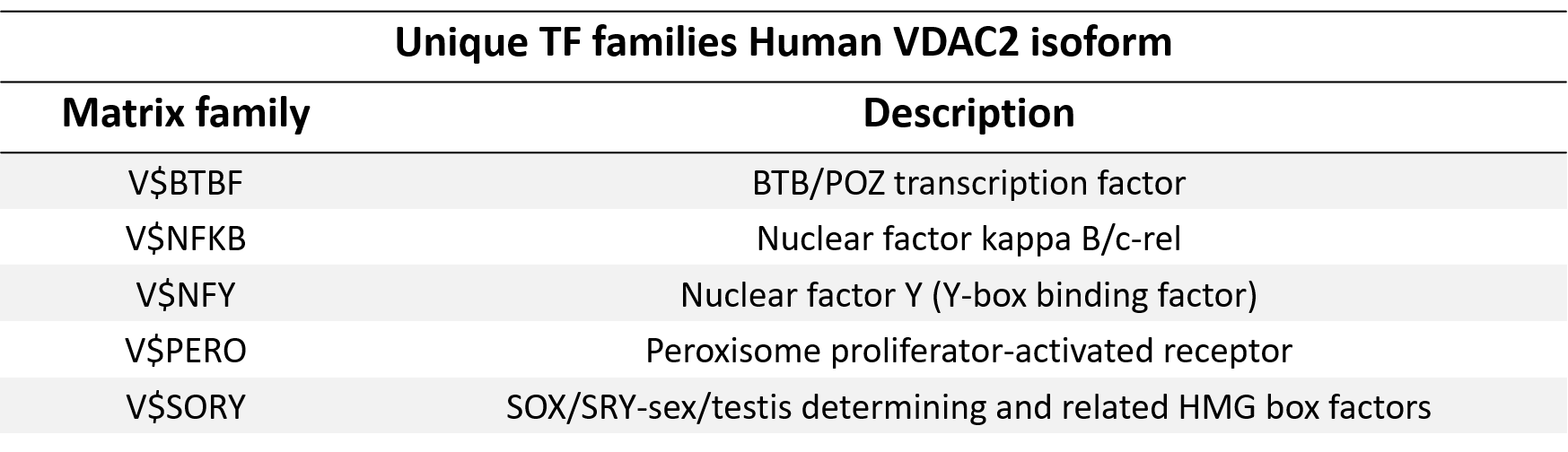


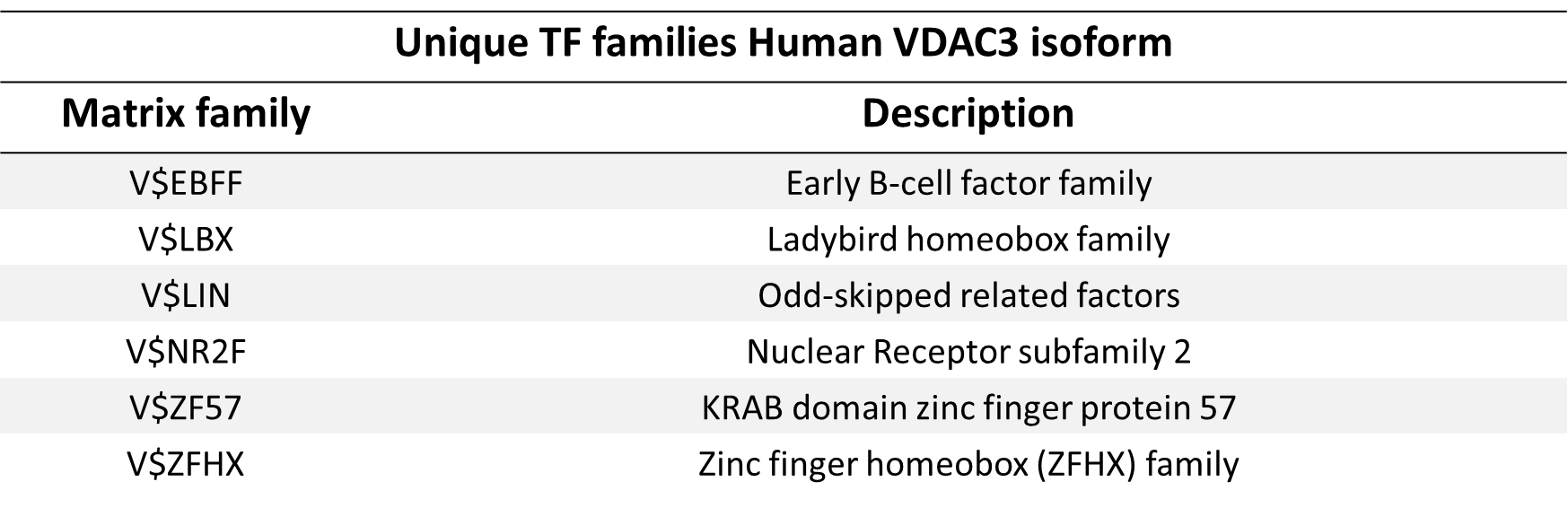


**Table S3.** **Matrix family and the description of the unique TFBS families in human VDAC3 promoter.**

**Table S4. Matrix family and the description of the unique TFBS families in mouse VDAC1 promoter.**


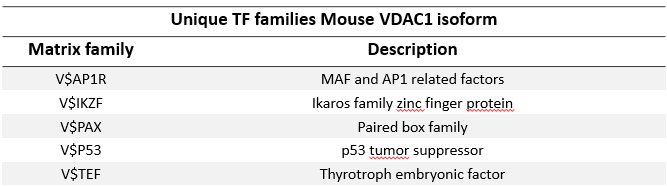


**Table S5. Matrix family and the description of the unique TFBS families in mouse VDAC2 promoter.**


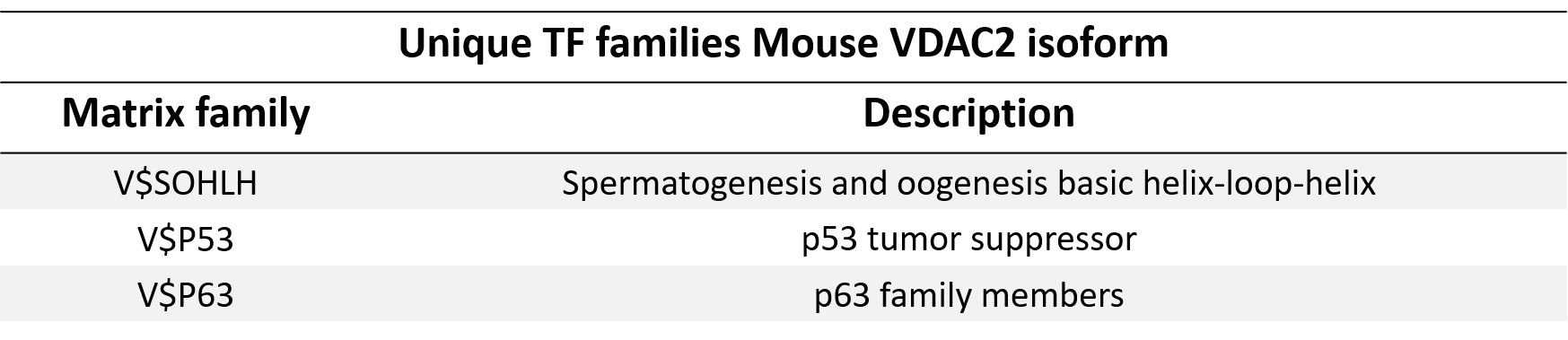


**Table S6. Matrix family and the description of the unique TFBS families in mouse VDAC3 promoter.**


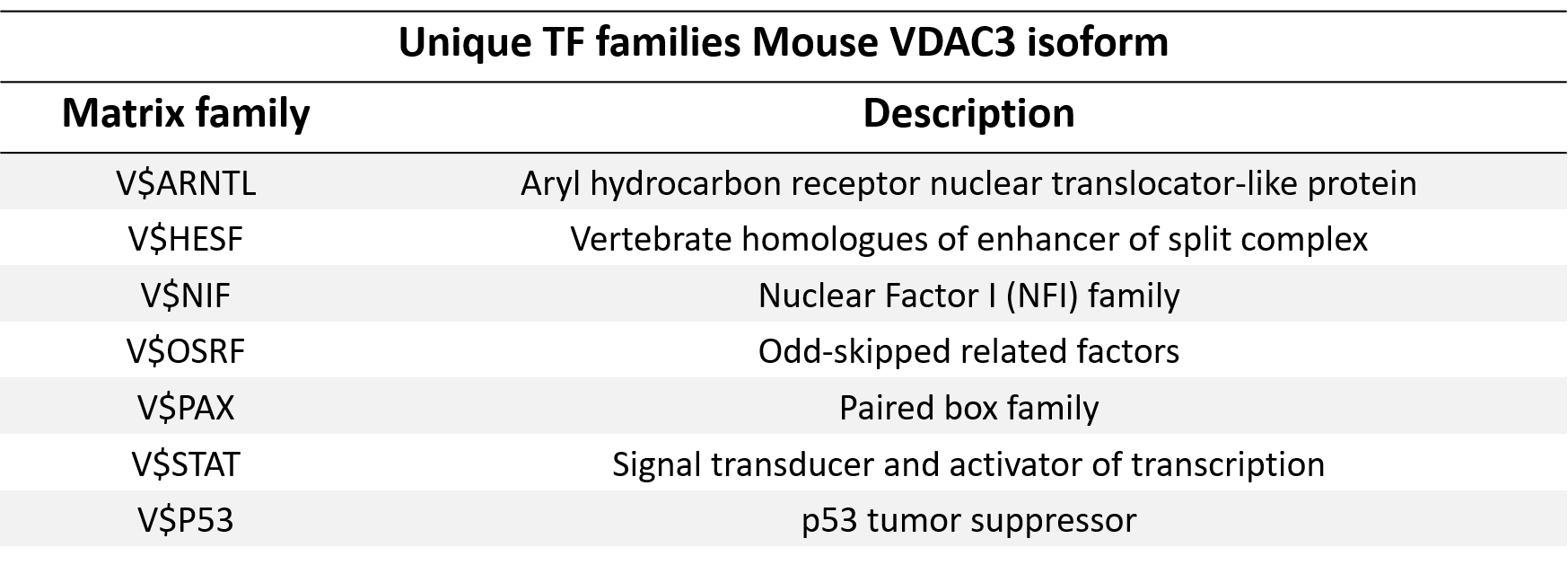

Supplement: Supplementary file 1 [file Table_1.DOCX]
